# Supplementary material for: Reconfigurable image processing metasurfaces with phase-change materials
Source: Nat Commun. 2024 May 27;15:4483. doi: 10.1038/s41467-024-48783-3 (PMC11130277; doi:10.1038/s41467-024-48783-3)
Supplement: Supplementary file 1 — Supplementary Information [file 41467_2024_48783_MOESM1_ESM.pdf]

Table of Contents

Section S1. Optical Characterization

Section S2. Additional Numerical Data

Section S3. Additional Experimental Data

Section S4. Calculated Edge-Detected Images for different input polarizations

Section S1. Optical Characterization

The measurements shown in Figs. 2-5 of the main paper were performed with the custom-built setups shown in Figure S1. In all measurements, the sample was attached to a thin ceramic heater (Thorlabs, HT10KR1) with a thermal tape (Thorlabs, TCDT1). The temperature of the heater was increased by progressively increasing an electrical current fed to the heater via a temperature controller (Thorlabs, TC300). The temperature of the sample was monitored by both a platinum-resistance temperature detector (Thorlabs, TH100PT) attached on the silicon side of the metasurface and a thermal camera. In the measurements with the thermal camera, it was assumed that the metasurface emissivity was 0.5. The heater was placed on two independent rotation stages: a motorized stage (Thorlabs, HDR50) to control the polar angle θ and a manual stage to control the azimuthal angle ϕ.

The normal-incidence measurements shown in Fig. 2 were acquired with the setup shown in Fig. S1a. A collimated broadband lamp was weakly focused on the metasurface with a lens (L1, focal length = 20 cm), and the beam transmitted through the sample was collected by an identical lens (L2) and sent onto the input slit of a near-infrared spectrometer (Ocean Optics, NIRQuest 512). The measurements in Figs. 2d were obtained by slowly increasing the current fed to the heater, and by continuously recording the transmission spectra and the device temperature. After the temperature reached a value of approximately 90° C, the current fed to the heater was turned off, and the transmission spectrum of the metasurface was continuously recorded as the sample cooled down to room temperature (Fig. 2e).

The angle-dependent measurements shown in Fig. 3 were performed with the same setup. A broadband supercontinuum laser (NKT, Fianium FIU-15) filtered via a tunable narrowband filter (Photon, LLTF Contrast) was used as a source. The linewidth of the filtered laser was approximately 5 nm. A fraction of the laser power was extracted with a beam-splitter (BS) and sent to a reference germanium power meter (Det1). Using the same lenses as in the previous measurements, the laser was weakly focused on the metasurface, and the transmitted signal was collected and re-collimated on the other side of the sample. The power level transmitted through the samples was recorded with another identical germanium power meter (Det2). A linear polarizer (LP1) placed before the beam-splitter was used to polarize the incoming beam along either x or y, which correspond, respectively, to p- and s-polarization for any value of θ and ϕ. A second linear polarizer (LP2) was used to select the output polarization. The transmission amplitudes shown in Figs. 3a were obtained with an automated procedure where the temperature was slowly increased in small steps and, after achieving a thermal steady state, the angle θ was swept, and the powers read with the power meters Det1 and Det2 were recorded. An additional reference measurement, performed without the sample, allowed obtaining the absolute transmission level, and to account for any potential fluctuation of the laser power.

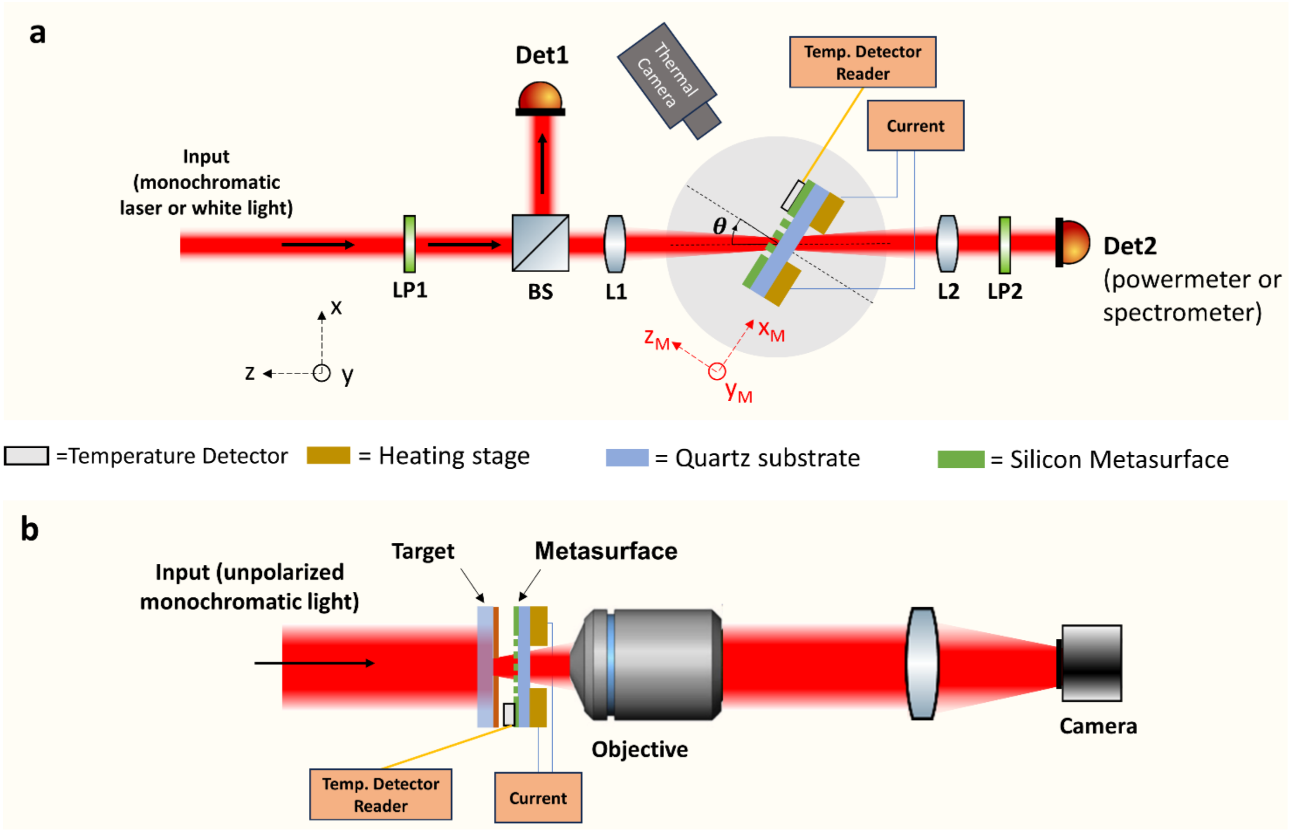

Figure S1. (a) Setup used for the angle- and temperature-dependent transmission measurements. (b) Setup used for the imaging experiments. Additional details in the text.

The imaging experiments shown in Figs. 4 and 5 of the main paper were performed with the setup illustrated in Fig. 4a and shown in more details in Fig. S1b. The illumination was provided by the same filtered supercontinuum source used in the setup described in the previous paragraph. Test input images were created by illuminating an amplitude mask with a collimated unpolarized light at a wavelength of 1672 nm. The mask was created by etching desired shapes onto a 200 nm thick layer of chromium deposited on a glass substrate. The image scattered by the target was collected with a NIR objective (Mitutoyo, 50X, NA = 0.42) and relayed onto a near-infrared camera (Ophir) with a f = 15 cm tube lens. The metasurface was mounted on the heating stage used for the measurements in Figs. 2 and 3 and placed between the mask and the objective. The heating stage was placed on a flip mount, allowing us to relay onto the camera either the unfiltered input image (when the metasurface is removed) or the input image filtered by the metasurface.

Section S2. Additional Numerical Data

In Figure 1d of the main paper we displayed the calculated amplitude of the s-polarized transfer functions of the metasurfaces, assuming that the VO_2 is either in the insulating (Fig. 1d, top) or metallic (Fig. 1d, bottom) phase. In Fig. S2, we show the calculated full angle-dependent complex

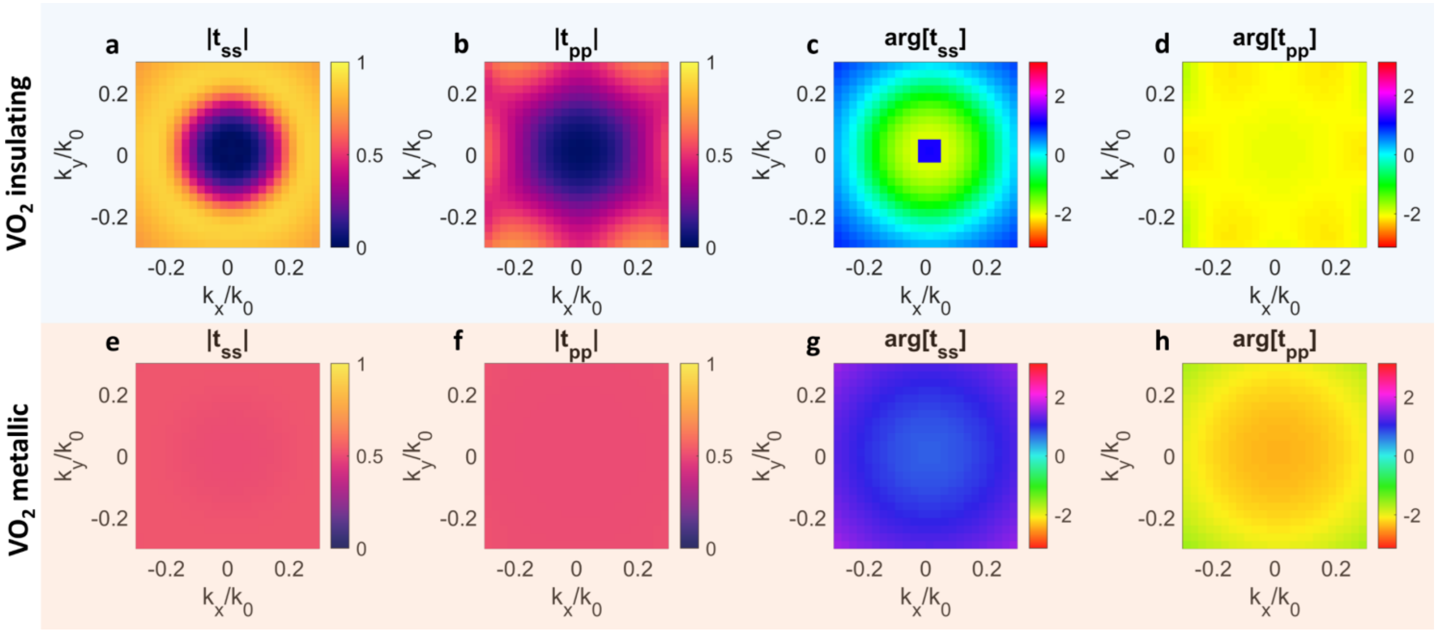

Figure S2. (a-d) Complex co-polarized transfer functions, for s polarization (t_{ss}, panels a and c) and p polarization (t_{pp}, panels b and d), calculated assuming that the VO_2 in the insulating phase. (e-h) Same as in panels a-d, but assuming that the VO_2 is in the metallic phase.

transfer functions for both s- and p-polarization and for both phases of VO_2. When the VO_2 is in the insulating phase, the amplitude of the transfer function displays the required Laplacian behavior for both s (Fig. S2a) and p polarization (Fig. S2b), i.e. the transmission is almost zero at normal incidence ($k_x = k_y = 0$) and it increases monotonically as a function of the in-plane wave vector $k_\parallel \equiv \sqrt{k_x^2 + k_y^2}$. Moreover, the transfer functions feature an almost perfect azimuthal isotropy within a numerical aperture of NA = 0.25. When the VO_2 is in the metallic phase, the amplitudes of both transfer functions (Figs. S2e and S2f) feature an almost flat profile with an approximately constant value close to 0.5. The corresponding phases of the transfer functions (Figs. S2(c-d) and S2(g-h)) are almost constant within the angular range of interest, as required by either the Laplacian or identity operation.

In Fig. S3 we show the electric field, magnetic field and power flow when the metasurface is excited at normal incidence, assuming that the VO_2 is either in the insulating (Figs. S3(a-c)) and metallic (Figs. S3(d-e)) phases.

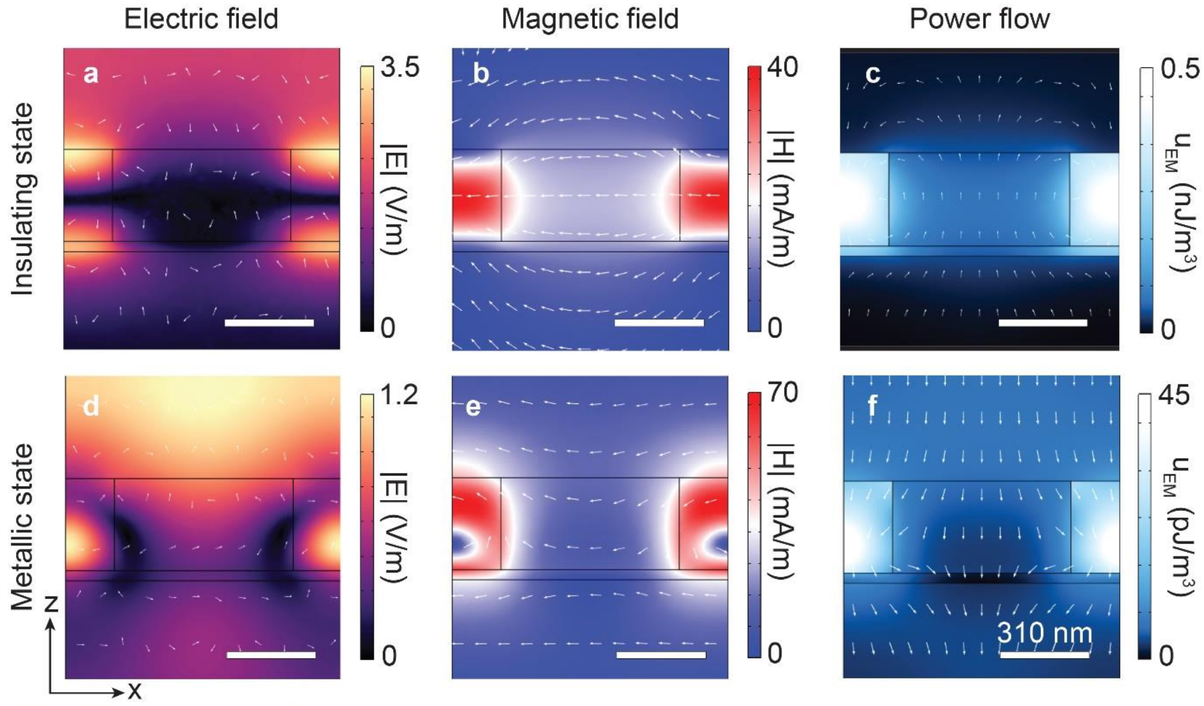

Figure S3. (a-c) Calculated electric field, magnetic field, and power flow density within the metasurface in the xz plane, for a normal-incidence wave at 1665 nm, and assuming that the VO_2 in the insulating phase. (d-f) Same as in panels a-c, but assuming that the VO_2 is in the metallic phase.

Section S3. Additional Experimental Data

In Fig. 4 of the main paper, we showed how the output image processed by the metasurface changes as a function of the temperature. In the main text we showed only a few select values of temperatures due to space constraints. In Fig. S4 we show the experimental data corresponding to all the temperatures acquired for the experiments shown in Fig. 4 of the main text. Moreover, Fig. S4 displays the color bar of each image (calibrated with the procedure described in the main text), which confirms that peak intensity of the edge-detected images remains constant for any temperature below the transition temperature of the VO_2. Similarly, the intensity of the bright-field images remains constant for any temperature above the transition temperature of the VO_2.

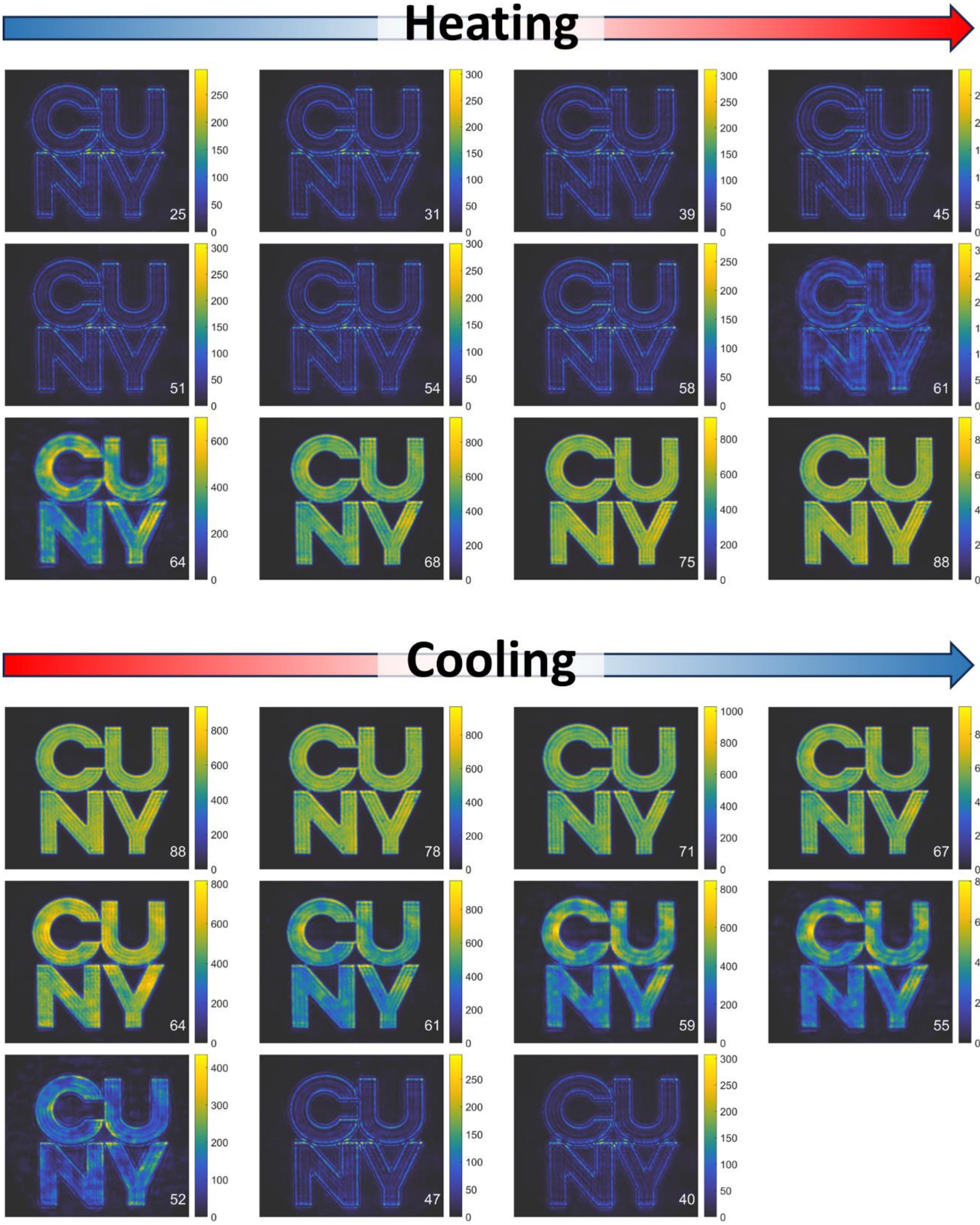

Figure S4. Extended dataset for the experiment shown in Fig. 4 of the main text, showing output images recorded at additional values of temperature.

Section S4. Calculated Edge-Detected Images for different input polarizations

In the experiments discussed in the main text, the input images were carried by unpolarized light. Given the asymmetric polarization response observed in Fig. 1e, one could wonder whether the quality of the processed images would be impacted when using polarized light in input. To assess this, numerically calculated the image processing imparted by the metasurface on input images with different polarizations. The calculations were done by following the formulas outlines in ref. [1], and by using the numerically calculated transfer functions shown in Fig. S2. The results of the calculations are shown in Fig. S5. We used an input image (Fig. S5a) similar to the one considered in the experiments in Fig. 4. The calculations confirm that, for either unpolarized (Fig. S5b), x-polarized (Fig. S5c) and y-polarized (Fig. S5d) illumination, all the edges of the input images are enhanced uniformly and independently of their orientation. Moreover, the peak intensities of the three output images are almost identical. This confirms that the small polarization asymmetry observed in Fig. 1e does not introduce any sizeable polarization dependence in the image processing.

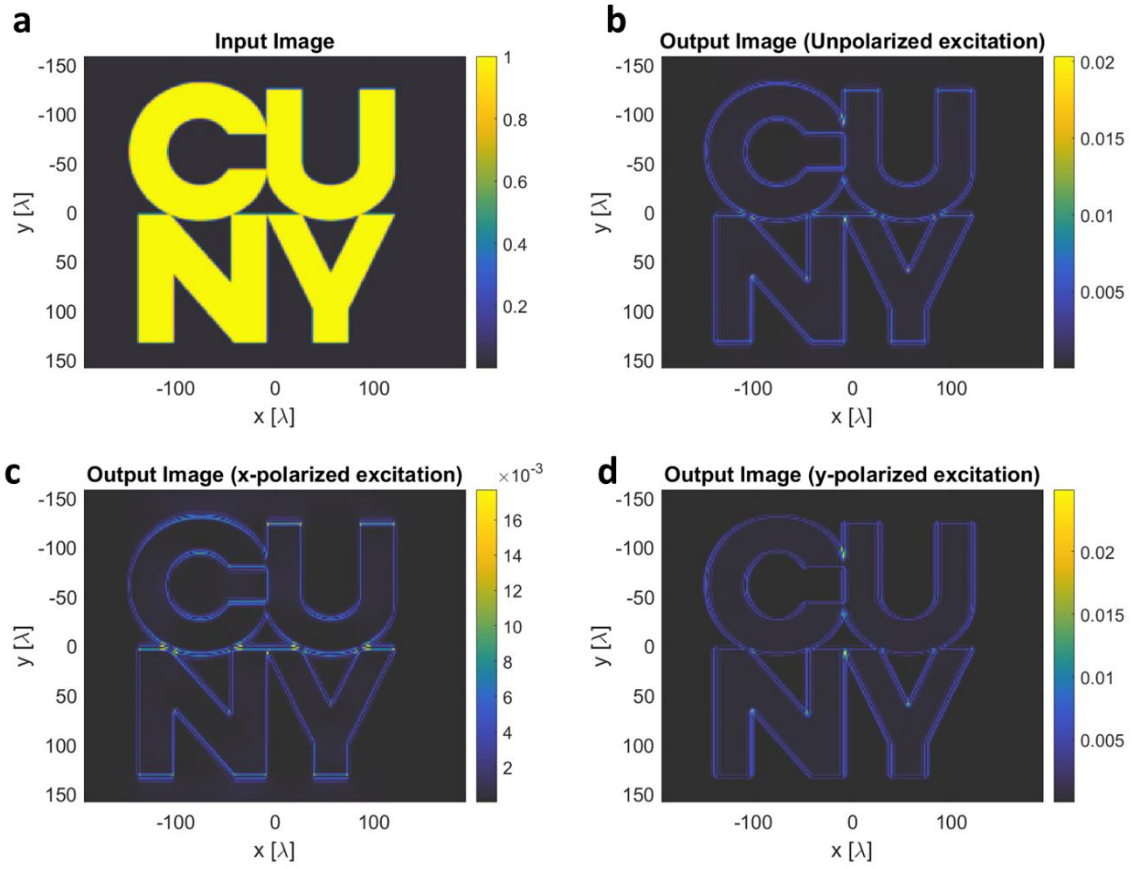

Figure S5. (a) Input image. (b-d) Output image calculated with the transfer functions shown in Fig. S2, and assuming that the input image is (b) unpolarized, (c) x-polarized, (d) y-polarized.
