# Supplementary material for: Reconfigurable image processing metasurfaces with phase-change materials
Source: Nat Commun. 2024 May 27;15:4483. doi: 10.1038/s41467-024-48783-3 (PMC11130277; doi:10.1038/s41467-024-48783-3)
Supplement: Supplementary file 3 — Description of Additional Supplementary Files [file 41467_2024_48783_MOESM3_ESM.docx]

**Supplementary Video 1**

Real-time image-processing experiment for a single heating-cooling cycle of the metasurface. At the beginning of the video, a constant current is applied to the heater. The video shows the picture captured by the camera as the metasurface is heated up. After the image processing has transitioned from edge detection to bright-field imaging, the current applied to the heater is turned off, and the metasurface is allowed to cool down to room temperature.
